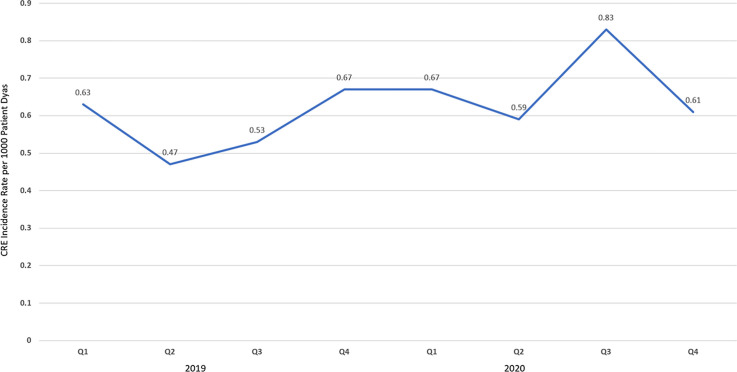# Impact of COVID-19 on Antimicrobial Use and Resistance in an Urban Safety-Net Community Hospital

**DOI:** 10.1017/ash.2021.32

**Published:** 2021-07-29

**Authors:** Alfredo Mena Lora, Ella Li, Fischer Herald, Nichelle Simpkins, Candice Krill, Eden Takhsh, Rodrigo Burgos, John Marchionne, Mirza Ali, Sherrie Spencer, Susan Bleasdale, Scott Borgetti

## Abstract

**Background:** The disease caused by SARS-CoV-2, COVID-19, has caused a pandemic leading to strained healthcare systems worldwide and an unprecedented public health crisis. Lower respiratory tract infections (LRTIs) and hypoxia caused by COVID-19 has led to an increase in hospitalizations. We sought to define the impact of COVID-19 on antimicrobial use and antimicrobial resistance (AMR) in an urban safety-net community hospital. **Methods:** Retrospective review of antimicrobial use and AMR in a 151-bed urban community hospital. Antimicrobial use was calculated in days of therapy per 1,000 patient days (DOT/1,000 PD) for ceftriaxone, piperacillin-tazobactam and meropenem during 2019 and 2020. Ceftriaxone, piperacillin-tazobactam and meropenem were reviewed for calendar year 2019 and 2020. AMR was assessed by comparing the carbapenem resistant Enterobacteriaceae (CRE) infection incidence rate per 1,000 patient days between 2019 and 2020. **Results:** The average quarterly DOT/1,000 PD increased from 359.5 in 2019 to 394.25 in 2020, with the highest increase in the second and fourth quarters of 2020, which temporarily correspond to the first and second waves of COVID-19. Ceftriaxone and meropenem use increased during the first and second waves of COVID-19. Piperacillin-tazobactam use increased during the first wave and declined thereafter (Figure 1). Rates of CRE increased from a quarterly average of 0.57 to 0.68 (Figure 2). **Conclusions:** Antimicrobial pressure increased during the first and second waves of COVID-19. Ceftriaxone was the most commonly used antimicrobial, reflecting internal guidelines and ASP interventions. CRE rates increased during COVID-19. This finding may be due to an overall increase in antimicrobial pressure in the community and in critically ill patients. Antibiotics are a precious resource, and antimicrobial stewardship remains important during the COVID-19 pandemic. Appropriate use of antimicrobials is critical to preventing AMR.

**Funding:** No

**Disclosures:** None

**Figure 1.**
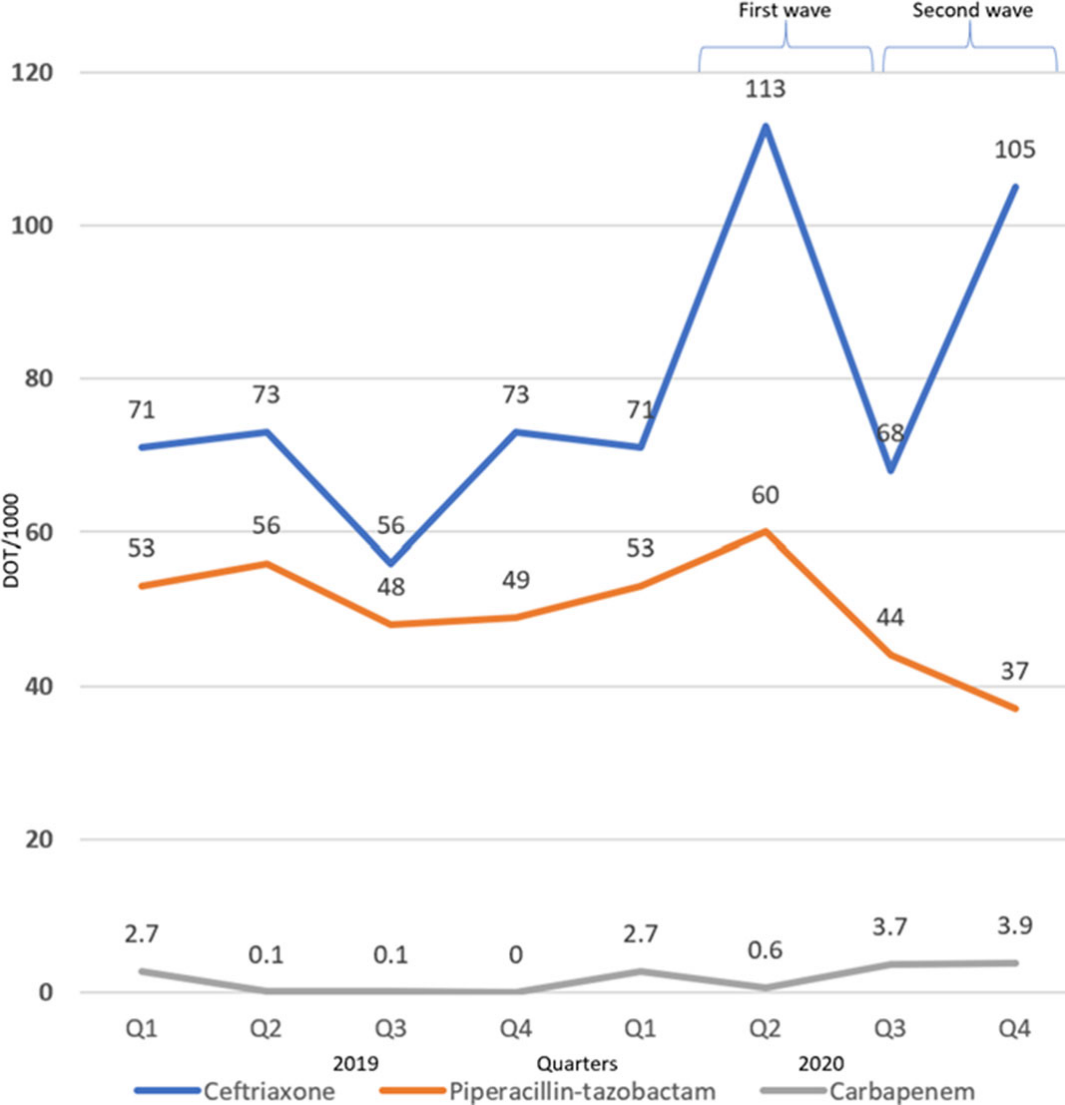

**Figure 2.**